# Minimal transmission of SARS-CoV-2 from paediatric COVID-19 cases in primary schools, Norway, August to November 2020

**DOI:** 10.2807/1560-7917.ES.2020.26.1.2002011

**Published:** 2021-01-07

**Authors:** Lin T Brandal, Trine S Ofitserova, Hinta Meijerink, Rikard Rykkvin, Hilde M Lund, Olav Hungnes, Margrethe Greve-Isdahl, Karoline Bragstad, Karin Nygård, Brita A Winje

**Affiliations:** 1 Norwegian Institute of Public Health, Oslo, Norway; 2 European Programme for Public Health Microbiology Training (EUPHEM), European Centre for Disease Prevention and Control, (ECDC), Stockholm, Sweden

**Keywords:** COVID-19, SARS-CoV-2, children 5–13 years, school, transmission, school-related contacts

## Abstract

An intense debate on school closures to control the COVID-19 pandemic is ongoing in Europe. We prospectively examined transmission of SARS-CoV-2 from confirmed paediatric cases in Norwegian primary schools between August and November 2020. All in-school contacts were systematically tested twice during their quarantine period. With preventive measures implemented in schools, we found minimal child-to-child (0.9%, 2/234) and child-to-adult (1.7%, 1/58) transmission, supporting that under 14 year olds are not the drivers of SARS-CoV-2 transmission.

Since summer 2020, a considerable increase in coronavirus disease (COVID-19) infections has been reported across Europe [1], including in Norway [2]. A better understanding of children’s role in transmission of severe acute respiratory syndrome coronavirus 2 (SARS-CoV-2) in school settings is urgently needed. Although several studies have reported limited transmission of SARS-CoV-2 among children in school settings [3-7], few have comprised systematic testing of contacts, including asymptomatic contacts. We aimed to examine transmission of SARS-CoV-2 from confirmed paediatric COVID-19 cases in primary schools in Norway by systematically testing all contacts within the school twice during their quarantine period.

## Identification of paediatric COVID-19 cases in primary schools and their contacts

**Figure 1:**
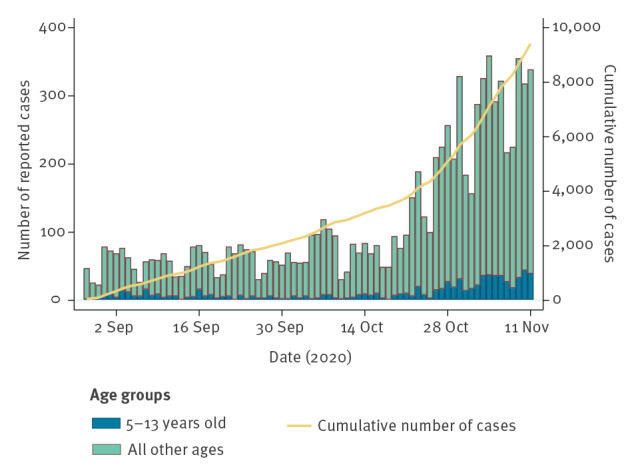
Laboratory-confirmed COVID-19 cases by date of sampling and age, Oslo and Viken counties, Norway, 28 August–11 November 2020 (n = 9,416)

Oslo and Viken were the counties in Norway with the highest 14-day incidence of COVID-19, ranging from 19.3 to 94.9 cases per 100,000 inhabitants in weeks 36 to 46 2020 [2]. During our observation period, from 28 August 2020 to 11 November 2020, the number of confirmed cases increased in these counties, including among children 5–13 years old (Figure 1).

COVID-19: coronavirus disease.

Source: Norwegian Surveillance System for Communicable Diseases (MSIS).

**Figure 2:**
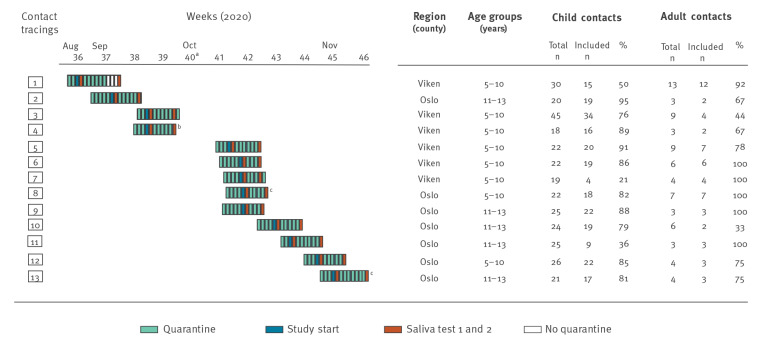
Overview of included COVID-19 contact tracings in schools, Oslo and Viken counties, Norway, 28 August–11 November 2020 (n = 13)

An index case was defined as a case aged 5–13 years in Oslo or Viken county with PCR-confirmed SARS-CoV-2 infection, who had attended school within 48 hours before symptom onset or date of sampling. We prospectively included contact tracings around 13 index cases. For each index case, public health officials identified exposed child and adult school contacts (Figure 2). All consenting cases and contacts delivered two self-collected saliva samples; the first was collected as soon as possible after they were identified, and the second was collected at the end of their 10-day quarantine period (Figure 2). We excluded contact tracings with adult COVID-19 index cases.

COVID-19: coronavirus disease.

^a^ Schools were closed due to autumn break in week 40 (28 September–2 October 2020).

^b^ The first saliva samples were taken from the index case twice, with 3 days between the two; the second of these was positive. There were 6 days between the original nasopharyngeal sample and the first positive saliva sample. The third and last saliva sample was taken at the end of the case’s 10-day quarantine.

^c^ One or two primary cases (school contacts who tested positive for SARS-CoV-2 in the first saliva sample) were detected in the contact tracing.

A primary case was defined as a school contact who tested positive for SARS-CoV-2 in the first saliva sample. A secondary case was defined as a school contact who tested positive for SARS-CoV-2 in the second saliva sample, following a first negative test.

Study invitations were sent to adult contacts (staff) and parents of child contacts through the school’s digital communication platform. We distributed equipment and detailed instructions for self-collection of two saliva samples to consenting participants on the same day. The first saliva sample was taken the following morning before eating, drinking or brushing teeth; the second saliva sample was taken at the end of the quarantine period with the same instructions (Figure 2). The participants added viral transport media to the saliva sample. Each saliva sample was analysed for SARS-CoV-2 using PCR (RdRp gene) [8] at the National Reference Laboratory of influenza and coronaviruses with outbreak potential (including SARS-CoV-2) at the Department of Virology at the Norwegian Institute of Public Health in Oslo.

## Minimal transmission of SARS-CoV-2 in primary schools

Thirteen contact tracings from primary schools were included: eight in the age group 5–10 years old (grades 1–4) and five in the age group 11–13 years old (grades 5–7) (Figure 2). A total of 13 index cases and 292 school contacts participated in the study. In Contact Tracing 7, the index case did not consent to participate and saliva samples from this individual could not be collected. In Contact Tracing 8, two index cases were included, and in all the remaining 11 contact tracings one index case was included. Four of the 13 index cases had attended school with mild symptoms (in Contact Tracings 2, 3, 4 and 13); however, among these four index cases’ contacts, only two primary cases (both in Contact Tracing 13) and no secondary case was identified. The remaining index cases were asymptomatic while attending school. All index cases, except one, had household members who were diagnosed with COVID-19 before the index cases themselves tested positive.

Among the 234 child contacts that were tested for SARS-CoV-2, two primary cases (0.9%) and no secondary cases were identified, and among the 58 adult contacts one primary case (1.7%) and no secondary cases were detected (Table 1). The three primary cases were identified in two different contact tracings (Contact Tracings 8 and 13, Figure 2).

**Table 1:** Outcome of contact tracings in schools from confirmed paediatric COVID-19 cases, Oslo and Viken counties, Norway, 28 August–11 November 2020

Age group (years)	Contact tracings	Index cases	Index likely infected in household	Asymptomatic index cases	Included contacts		Primary cases^a^		Secondary cases^b^	
					Children	Adults	Children	Adults	Children	Adults
5–10	8	8^c^	7^d^	6	148	45	1	0	0	0
11–13	5	5	5	3	86	13	1	1	0	0
**Total**	**13**	**13**	**12**	**9^e^**	**234**	**58**	**2**	**1**	**0**	**0**

COVID-19: coronavirus disease.

^a^ Primary cases were defined as school contacts identified as SARS-CoV-2 positive in the first saliva sample.

^b^ Secondary cases were defined as school contacts identified as SARS-CoV-2 positive in the second saliva sample, following a negative first test.

^c^ In one contact tracing (no. 8), two index cases were present at the start of the study and both were included. They had overlapping school-related contacts. In one contact tracing (no. 7), the index case did not consent to participate.

^d^ One index case did not have a household member diagnosed with COVID-19 prior to their own diagnosis, but had visited a friend’s household with adult COVID-19 cases before testing positive for SARS-CoV-2.

^e^ Four index cases attended school while having mild symptoms (two in the age group 5–10 years and two in the age group 11–13 years).

The median number of contacts per index was 19 children (interquartile range (IQR): 16–21) and three adults (IQR: 2.5–6.5). We observed a high participation rate of 73% (234/319) (IQR: 63–89%) among child contacts and 78% (58/74) (IQR: 67–100%) among adult contacts (Figure 2, Table 1).

## Self-collected saliva for detection of SARS-CoV-2 in index cases and their school contacts

SARS-CoV-2 was confirmed in the self-collected saliva samples of 11 of the 13 index cases [8] (Table 2). In two index cases the saliva tests were negative. One child had drunk a glass of milk before the saliva collection. The other had a weakly positive nasopharyngeal sample (SARS-CoV-2 PCR cycle threshold (Ct) value of 35.73) 2 days before the saliva collection, which may explain the discrepant results. The median was 3 days (range: 2–6) between the confirmed nasopharyngeal SARS-CoV-2 sample and the first saliva sample, and 6 days (range: 3–10) between the first and the second saliva samples.

**Table 2:** Detection of SARS-CoV-2 in saliva samples from paediatric COVID-19 index cases and their school contacts, Oslo and Viken counties, Norway, 28 August–11 November 2020

Contact tracings	Age (years)	Index/contact	Days between nasopharyngeal sample and saliva 1^a^	Saliva 1 (Ct value)		Days between saliva 1 and saliva 2	Saliva 2 (Ct value)	
				nCoV_IP2	nCoV_IP4		nCoV_IP2	nCoV_IP4
1	7	Index	3	24.56	23.24	10	Neg	Neg
2	12	Index	5	26.71	24.96	6	34.92	33.8
3	9	Index	NA^b^	NA^b^	NA^b^	8^c^	29.44	27.84
4	7	Index	6	38.27^d^	32.97^d^	3	Neg	Neg
5	7	Index	2	24.26	22.39	7	34.50	30.78
6^e^	6	Index	5	Neg	Neg	7	Neg	Neg
7^f^	NA	Index	NA^b^	NA^b^	NA^b^	NA^b^	NA^b^	NA^b^
8^g^	8	Index	5	21.17	20.47	5	26.64	25.15
	8	Index	3	24.86	23.93	5	27.49	25.70
	8	Contact (child)	NA^b^	29.55	28.56	5	18.58	17.40
9	12	Index	5	19.05	18.71	5	20.06	18.72
10^h^	11	Index	4	Neg	Neg	6	Neg	Neg
11	11	Index	3	26.17	25.45	7	31.25	30.92
12	6	Index	3	17.47	16.75	6	31.25	30.92
13	11	Index	3	23.22	23.04	7	28.89	27.39
	11	Contact (child)	NA^b^	39.17	35.42	7	23.49	22.01
	26	Contact (adult)	NA^b^	11.08	10.66	7	29.32	28.15

COVID-19: coronavirus disease; Ct: cycle threshold; NA: not applicable; Neg: negative.

^a^ Nasopharyngeal sample is routinely used for detection of SARS-CoV-2 by PCR in medical microbiological laboratories in Norway.

^b^ Not applicable, since no saliva sample was collected or no nasopharyngeal sample was taken before the saliva test.

^c^ Number of days between nasopharyngeal sampling for routine detection of SARS-CoV-2 by PCR and sampling of saliva sample 2. The first saliva sample was not collected from this index case.

^d^ The first saliva sample was taken twice from this index case. The last saliva sample was taken at the end of their 10-day quarantine.

^e^ Index case drank milk before collection of saliva sample 1.

^f^ In Contact Tracing 7, the index case did not consent to participate and thus no saliva samples were collected from this case.

^g^ Two index cases were reported from the same class, with overlapping school-related contacts. Both were included in this contact tracing.

^h^ The nasopharyngeal sample was positive for SARS-CoV-2 with a Ct value of 35.73, 4 days before sampling of saliva 1.

‘Saliva 1’ indicates the first saliva sample, taken as soon as the index case or case contact was identified, and ‘saliva 2’ indicates the second saliva sample, taken at the end of their 10-day quarantine.

All contacts, except three, tested negative in both saliva samples.

## Ethical statement

The Regional Committees for Medical and Health Research Ethics in Norway approved this study (reference 151649). It complies with the European Union general data protection regulation (GDPR) requirements. Informed consent was obtained from adult contacts and parents of child contacts.

## Discussion

This prospective study shows that transmission of SARS-CoV-2 from children under 14 years of age was minimal in primary schools in Oslo and Viken, the two Norwegian counties with the highest COVID-19 incidence and in which 35% of the Norwegian population resides. In a period of low to medium community transmission (a 14-day incidence of COVID-19 of < 150 cases per 100,000 inhabitants) [9], when symptomatic children were asked to stay home from school, there were < 1% SARS-CoV-2–positive test results among child contacts and < 2% positive results in adult contacts in 13 contract tracings in Norwegian primary schools. In addition, self-collection of saliva for SARS-CoV-2 detection was efficient and sensitive (85% (11/13); 95% confidence interval: 55–98).

Studies from several European countries have shown minimal transmission of SARS-CoV-2 from paediatric index cases in schools [3-7]. However, the majority of these studies did not consider asymptomatic infections and did not screen all contacts. Our study confirms and strengthens these data, as we found minimal transmission even with a prospective design and systematic testing of all contacts twice during quarantine.

Most of our index cases were asymptomatic and were tested for SARS-CoV-2 by PCR because they were contacts of positive household members, supporting that household transmission is a considerable source of SARS-CoV-2 infection in children [6,10]. Although the number of adult contacts was limited, we found SARS-CoV-2 only in one (1.7%) of the adult contacts. This supports findings in Sweden, the Netherlands and Norway that teachers are not at higher risk of COVID-19 compared with other professions [11-13].

Few studies have used saliva samples for detection of SARS-CoV-2 in children and results have been discrepant [14-16]. Our data indicate that saliva samples have a good sensitivity; however, providing clear instructions for correct saliva sampling is necessary. Self-collection of saliva has advantages compared with nasopharyngeal sampling; it is non-invasive, comfortable, easy, cheap and can be done without involvement of healthcare workers. Of note, the number of observations here are small and our data do not enable us to calculate the specificity of self-collection of saliva. Although self-collection of saliva is an attractive method, further studies are needed to accurately define its sensitivity and specificity.

Norwegian schools were closed during the first SARS-CoV-2 wave in spring 2020. After re-opening, all schools implemented infection prevention and control (IPC) measures based on national guidelines. These included strengthened hygiene measures, physical distancing and a clear message to stay home if symptomatic, even with mild symptoms [17]. Use of face masks is not recommended in schools in Norway. We found that with the IPC measures implemented there is low to no transmission from SARS-CoV-2–infected children in schools. This finding strengthens national guidelines to adjust IPC measures according to the community transmission level [18], rather than closing primary schools for on-site teaching. Results from other studies further strengthen this position. Small school outbreaks elsewhere in Europe have been controlled by IPC measures [5,6], whereas a lack of adequate IPC measures has resulted in a large COVID-19 school outbreak in Israel [19].

It is important to acknowledge that our results are valid for primary schools only, and not for secondary or high schools. SARS-CoV-2 transmission in those ≥ 14 years old needs to be further studied.

## Conclusions

Systematic tracing and testing of school contacts of paediatric COVID-19 cases showed minimal child-to-child and child-to-adult transmission in primary schools with implemented IPC measures. The results obtained during low to medium community transmission demonstrate the limited role of children in transmission of SARS-CoV-2 in school settings. This is an important finding in view of the ongoing discussions on school closures and use of quarantine for a large number of children. Strengthening of IPC measures in schools when community transmission levels increase could be an option.
